# Challenges in breast cancer screening and the role of the gynecologist: Number 16 – 2026

**DOI:** 10.61622/rbgo/2026FPS16

**Published:** 2026-10-13

**Authors:** Cynthia Mara Brito Lins Pereira, Felipe Pereira Zerwes, Guilherme Novita, Rosemar Macedo Sousa Rahal

**Affiliations:** Universidade Federal do Pará Belém PA Brazil Universidade Federal do Pará, Belém, PA, Brazil.; Pontifícia Universidade Católica do Rio Grande do Sul Escola de Medicina Porto Alegre RS Brazil Escola de Medicina, Pontifícia Universidade Católica do Rio Grande do Sul, Porto Alegre, RS, Brazil.; American Society of Breast Surgeons New York United States American Society of Breast Surgeons, New York, United States.; Universidade Federal de Goiás Goiás GO Brazil Universidade Federal de Goiás, Goiás, GO, Brazil.

## Key points

- Breast cancer screening offers consistent evidence of mortality reduction, particularly when it enables detection at early stages. Early diagnosis expands the options for less aggressive treatments, improves clinical outcomes, positively impacts survival, and contributes significantly to patients’ quality of life.
- Identifying high-risk women, including those with a significant family history or associated genetic mutations, is crucial for tailoring the starting age and frequency of screening. Adopting personalized protocols allows for the rational use of additional tests, promoting greater clinical effectiveness and better cost-effectiveness for the healthcare system. Magnetic resonance imaging (MRI) should be considered for high-risk patients and those with dense breasts; if MRI is unavailable, breast ultrasound can be used as an alternative.
- Despite proven benefits, screening is associated with limitations, such as false-positive results and overdiagnosis, which can lead to anxiety and unnecessary interventions. In this context, clear communication and shared decision-making are essential to ensure the patient understands the potential risks and benefits involved.
- Tomosynthesis (3D mammography), combined with supplemental breast MRI for extremely dense breasts, can enhance the benefits of early diagnosis but also increases the risk of false-positives. Further studies are needed to provide more long-term data and assist in clinical and policy discussions, particularly regarding patients with dense breasts.
- Breast cancer screening in Brazil still faces cultural, structural, and socioeconomic barriers, alongside regional inequalities.
- The gynecologist plays a central role as the entry point for preventive actions in women's health. These specialists are responsible for encouraging regular adherence to screening, provide guidance based on up-to-date scientific evidence, and coordinate care, ensuring timely referral and proper integration with the specialized care network.

## Recommendations

- The implementation and consolidation of organized mammography screening programs nationwide is strongly recommended. Efforts must be made to expand equitable access and reduce regional disparities, while also ensuring technical standardization, rigorous quality control, and continuous monitoring of care indicators.
- Recommendations regarding the starting age and frequency of screening should be harmonized and based on the best available scientific evidence. Guidelines must take into account the Brazilian epidemiological profile and be periodically reviewed as scientific knowledge evolves.
- Systematic assessment of risk factors during gynecological consultations is recommended. Women identified as high-risk should follow specific protocols involving the judicious use of additional tests, aiming to maximize benefits and minimize harm while maintaining cost-effectiveness.
- Clear, objective, and evidence-based communication regarding the benefits, limitations, and potential risks of screening, including the possibility of false positives and overdiagnosis, must be ensured. Encouraging shared decision-making is recommended to promote patient autonomy and informed understanding.
- The gynecologist plays a central role in promoting secondary prevention by encouraging regular adherence to screening and ensuring timely referral. Integrated action with the specialized care network is recommended to ensure continuity and quality of care.

## Background

Screening involves performing imaging tests on asymptomatic women from the general population with the aim of detecting subclinical lesions.^(1–3)^ Detecting breast cancer at an early stage offers a greater chance of cure and the possibility of less aggressive treatments. Despite these benefits, screening is also associated with potential adverse effects, which must be properly discussed with patients.^(4–6)^

It is worth noting that the first effective treatment for breast cancer, radical mastectomy, was described in 1894. Between 1900 and 1950, no new therapeutic modalities for the disease emerged. Nevertheless, a significant reduction in breast cancer mortality was observed (from approximately 80% to 50%). This is attributed to patients seeking treatment at earlier stages of the disease, validating the concept that early-stage detection offers a greater chance of cure.^(5)^

Despite this finding, population-based campaigns encouraging breast self-examination have shown no clinical benefit in population studies. While it is clearly essential to educate the public on the need to investigate palpable lesions, encouraging routine self-examination among asymptomatic women has been associated with more harm than good. Palpation can create a false sense of security regarding the absence of disease, in addition to increasing false-alarm rates and leading to unnecessary invasive procedures.

The first effective studies on mammographic screening were conducted in European countries and North America during the 1970s. In general, these studies compared two geographically similar populations. In one of them, asymptomatic women aged 40 to 70 were encouraged to undergo periodic mammography.^(1–3)^ The results of these studies are illustrated in figure 1.^(2)^

**Figure 1:**
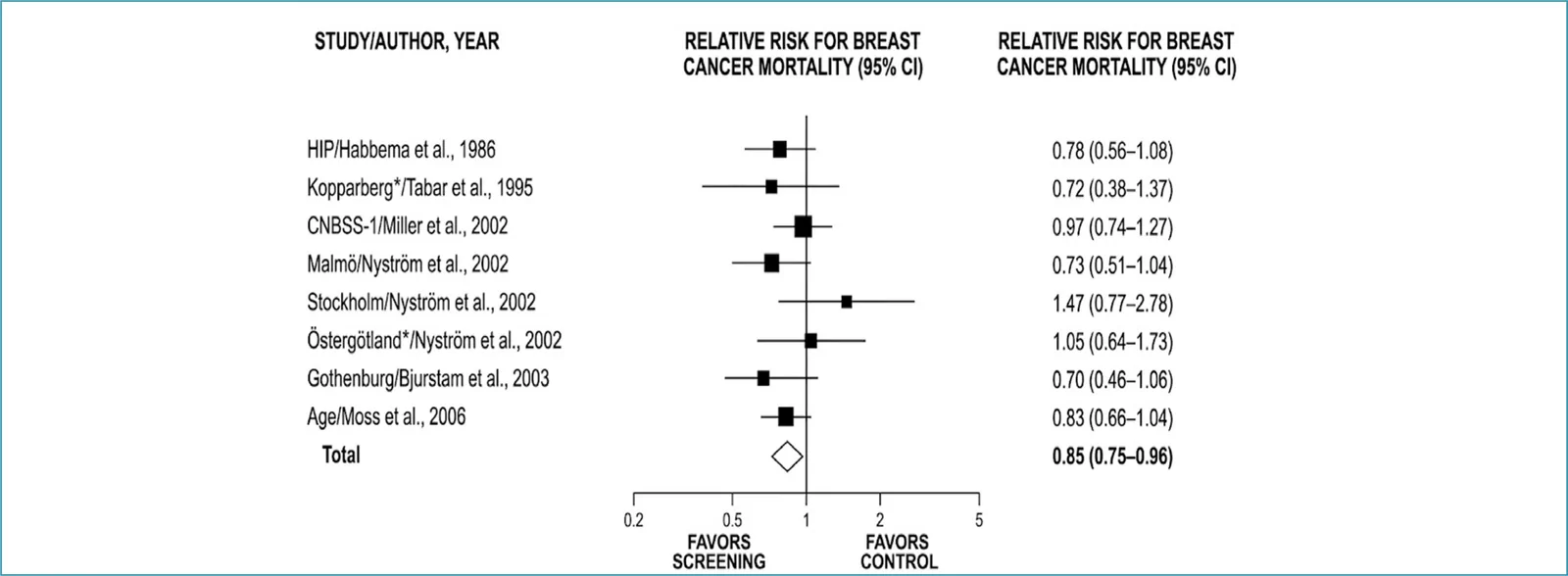
Population-based breast cancer screening studies demonstrating a 15% mortality reduction.

CI: confidence interval.

Source: Adapted from Nelson et al. (2009).^(2)^

A pooled analysis of these studies was conducted by several independent bodies, including the Cochrane Group, the Canadian Task Force, and the UK Task Force.^(1,2,7)^ The conclusions of these reviews consistently demonstrate that mammographic screening reduces breast cancer mortality by approximately 15% to 20%.^(1,2)^ The impact of early detection on mortality reduction is estimated to be comparable to the combined effect of all available treatments (surgery, radiotherapy, and systemic therapies),^(4)^ as shown in figure 2.^(2)^

**Figure 2:**
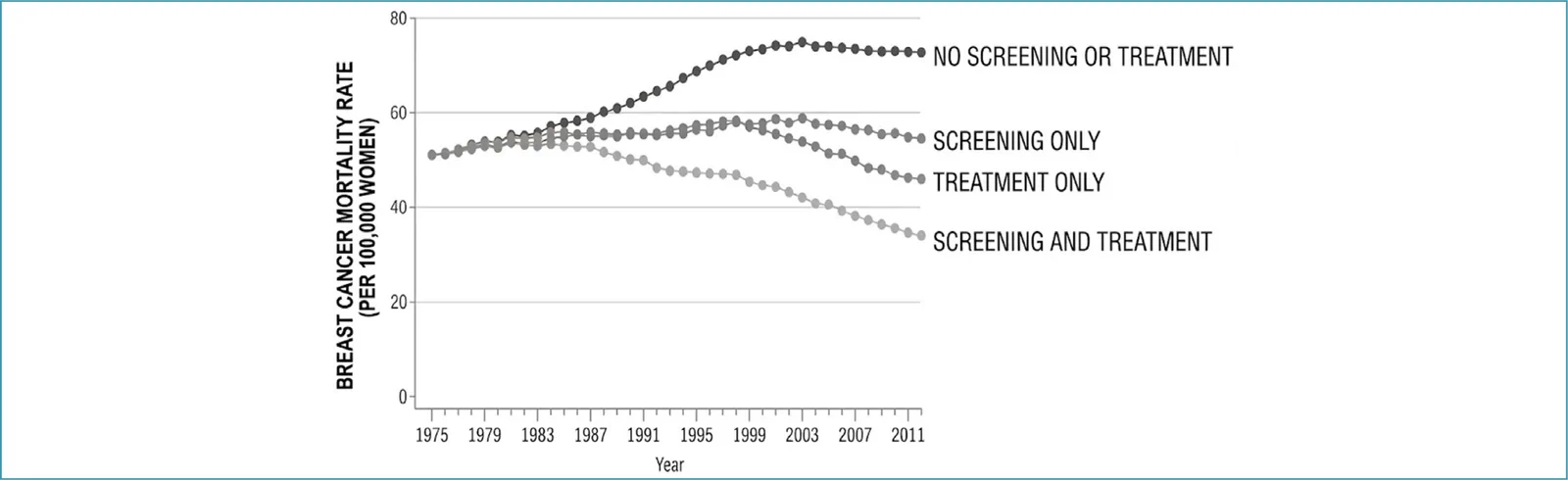
Estimated effectiveness of mammography screening compared to treatment

Source: Adapted from Nelson et al. (2009).^(2)^

Another relevant aspect is that tumors diagnosed at early stages require less pharmacological treatment, which reduces costs for the healthcare system and improves patients’ quality of life.^(4,8)^ Although studies demonstrate a reduction in mortality among women aged 40 to 70, screening is most effective in the 50–70 age group. Data analysis shows that older women experience a greater reduction in mortality, associated with lower rates of false-positive results and unnecessary biopsies, as presented in table 1.^(2,9)^

**Table 1:** Mammography screening results by age group

Age (years)	40–49	50–59	60–69	70–79	80–89
Reduction in mortality	15% (95% CI: 4%–25%)	14% (95% CI: 1%–25%)	32% (95% CI: 12%–46%)	NA	NA
Number needed to screen (NNS) to prevent 1 death	1,904	1,339	377	NA	NA
Number of patients to detect 1 cancer	556	294	200	154	143
Number of additional images to detect 1 cancer	47	22	14	10	8
Number of biopsies to detect 1 cancer	5	3	2	2	1.5

CI: confidence interval; NA: not applicable.

Source: Adapted from Nelson et al. (2009)^(2)^ and Glechner et al. (2023).^(9)^

These data indicate that screening is the most effective in women aged 60 to 69. However, this does not invalidate the benefit of mammography screening for younger women, particularly those in the 40–49 age group.^(9)^

## What do current guidelines recommend for breast cancer screening?

The vast majority of medical societies recommend periodic mammography for all women starting at age 40.^(10–12)^ Although most screening studies have included women up to age 70 or 75, screening is generally considered to require an individualized approach beyond that age.^(1,3)^ The most widely accepted recommendation is that women with a life expectancy of five years or more should continue to undergo periodic mammography.^(10)^ Screening intervals vary across guidelines. Most medical societies recommend annual mammography, while some groups suggest biennial intervals.^(1,12)^ Despite clear recommendations from medical societies regarding the need for annual exams between the ages of 40 and 75, various countries adopt different government guidelines.^(10–13)^

In Brazil, the Ministry of Health recommends biennial mammography screening for women aged 50 to 69. Similar guidelines are adopted in most European countries. These recommendations do not reflect superior outcomes in terms of mortality reduction but are primarily linked to budgetary constraints and the availability of public resources. There is no evidence supporting the benefit of the routine use of breast ultrasound or magnetic resonance imaging (MRI) in population-based breast cancer screening programs. Therefore, the inclusion of these exams is not systematically recommended, except in specific situations.^(7,11–14)^

## What are the controversies surrounding mammographic screening?

Despite consistent evidence of reduced mortality, mammographic screening faces criticism.^(2,15)^ Although many of these criticisms are unfounded, some issues warrant consideration. Key points of criticism include:

- False-positive results: abnormal findings leading to the need for additional tests, biopsies, or surgeries, without a confirmation of breast cancer.^(2,15)^
- Overdiagnosis: the detection of indolent neoplasms that would not manifest clinically during the patient's lifetime.^(15)^

Mammographic screening is not without limitations. In the BI-RADS™ 4 category, it is estimated that approximately 30% of biopsies confirm malignancy, implying that about 70% of the procedures are performed on benign lesions.^(2)^ Another relevant criticism concerns the interpretation of screening benefits, particularly due to the confusion between relative risk reduction and absolute risk reduction.^(9)^ The Canadian Task Force demonstrated that in the 40-49 age group, the absolute reduction in mortality is 0.05% (1 death per 313 women versus 1 death per 370), corresponding to a relative reduction of approximately 15%.^(9)^ Additionally, the economic impact of screening programs is a significant factor, largely due to false-positive results and overdiagnosis.^(2,15)^ This is a particularly sensitive issue, as population data indicate that the implementation of screening substantially increases the number of early-stage breast cancer diagnoses without a proportional reduction in advanced-stage cases, as illustrated in figure 3.^(5)^

**Figure 3:**
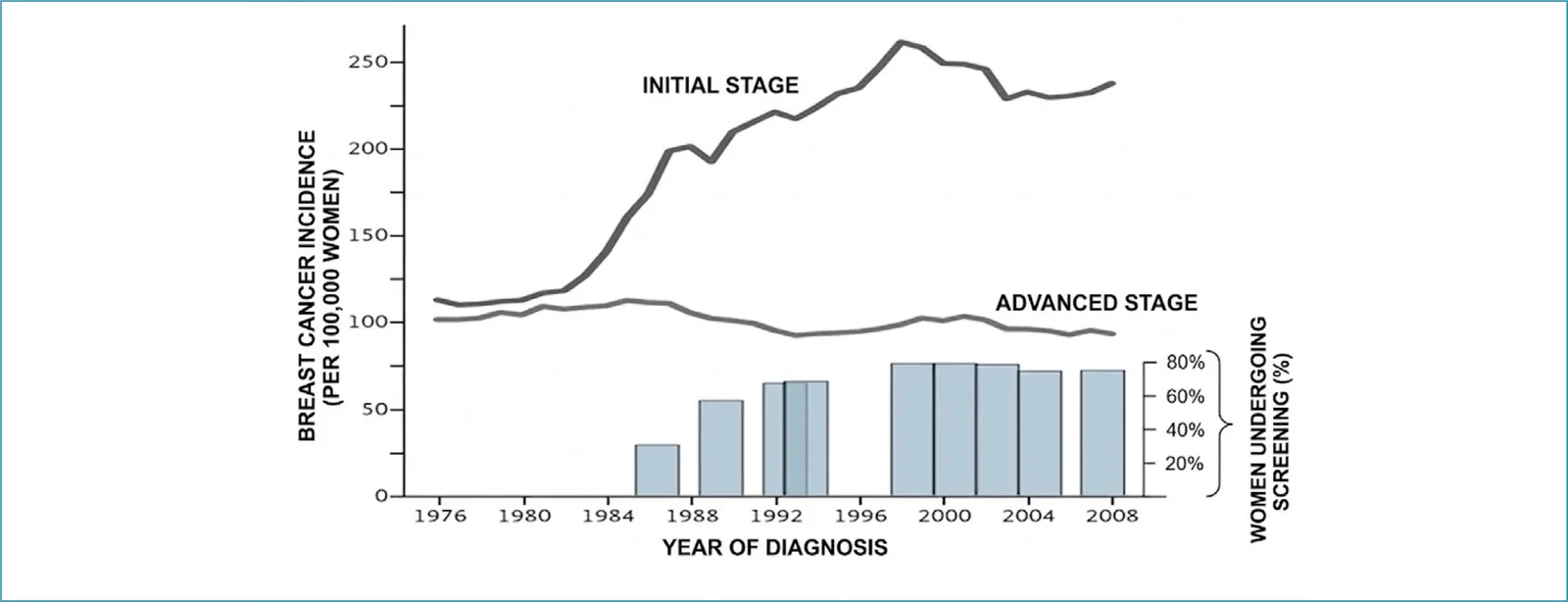
Population-level effect of implementing a mammography screening program, with an increase in early-stage cases alongside a stable number of advanced-stage cases.

Source: Adapted from Bleyer and Welch (2012).^(5)^

In light of the above, although mammography screening is not without limitations, it remains the most effective strategy for reducing breast cancer mortality in the general population. Clear and transparent communication regarding risks and benefits should always be part of the shared decision-making process with the patient. However, individual decisions should not invalidate population-level recommendations based on scientific evidence. Criticisms of screening programs are relevant and should guide the development of future studies. Nevertheless, the best evidence available to date, derived from randomized population-based studies, strongly supports maintaining current programs.^(1,2,7,15)^

Therefore, it is recommended that all asymptomatic women at average risk undergo annual mammography starting at age 40, provided they remain in good health.^(10–12)^

## What is the impact of mammography screening on breast cancer mortality?

Mammography screening is associated with a statistically significant reduction in breast cancer-specific mortality, particularly among women in the age groups with the highest incidence. Early detection, especially at early stages (stages 0 and I, according to the TNM classification), is linked to increased overall and cancer-specific survival, as well as a reduced need for more aggressive adjuvant systemic therapies. Identifying tumors at an early stage also enables breast-conserving surgical interventions, positively impacting oncological outcomes and quality of life.^(1,4,16)^

## What factors limit the effectiveness of screening in Brazil?

The effectiveness of screening in the Brazilian context is compromised by regional inequalities in the availability of mammography, insufficient coverage of the target population, the absence of structured, organized national programs, and variability in technical quality and exam standardization. Additionally, conflicting recommendations regarding the starting age and frequency of screening contribute to heterogeneity in clinical practice and hinder the establishment of uniform, evidence-based public policies.^(13,17)^

## How does risk stratification contribute to optimizing screening?

Risk stratification enables the identification of population subgroups at higher risk of developing breast cancer, including carriers of pathogenic variants in predisposition genes (such as BRCA1 and BRCA2), women with a significant family history, a history of high-risk breast lesions, and those with high breast density. For these groups, adopting tailored protocols, such as starting screening earlier and/or using complementary methods like MRI, can increase diagnostic sensitivity and improve the screening's risk-benefit ratio. Ultrasound may be considered in specific situations, particularly when MRI cannot be performed.^(14,18)^

In this context, risk prediction models serve as important tools for estimating individual risk and guiding the choice of follow-up strategy. Quantitative assessment should be specifically considered in cases involving significant family history, high-risk proliferative lesions, high breast density combined with other factors, or other elements suggesting a risk level above that of the general population.

Among the available models, CanRisk, based on the BOADICEA model, stands out for integrating family history, clinical factors, genetic information, and other risk factors, while also estimating the likelihood of carrying pathogenic variants in predisposition genes. Thus, it is particularly useful for women with a family history suggestive of hereditary predisposition or those requiring evaluation for genetic counseling. Although validation data specific to the Brazilian population remain limited, its scope makes it a relevant tool for individual risk assessment.^(19,20)^

Other models may also be used depending on the clinical profile. The Tyrer-Cuzick (IBIS) model incorporates family, reproductive, and clinical factors, as well as breast density, making it useful for estimating risk in women with multiple associated factors.^(21)^. The Gail model, in turn, estimates absolute breast cancer risk over five years and across a lifetime. It is best suited for women without a strong suspicion of hereditary syndrome, as it has limitations regarding the assessment of complex family histories and carriers of pathogenic variants.^(22)^

## What are the potential harms associated with screening?

Although screening offers proven benefits in terms of mortality reduction, it is associated with potential adverse events, including false-positive results, the need for additional follow-up tests, benign biopsies, and the overdiagnosis of lesions with low potential for clinical progression. These factors can lead to psychological impact, increased anxiety, and higher healthcare costs. A careful assessment of the risk-benefit ratio and the implementation of shared decision-making are essential to mitigate such effects.^(2)^

## What is the gynecologist's role in the effective implementation of screening?

Gynecologists play a central role in the secondary prevention of breast cancer. They are responsible for conducting individualized assessment of risk factors, providing guidance based on the best available scientific evidence, and encouraging regular adherence to screening. Furthermore, they act as care coordinators, ensuring timely referral for further diagnostic investigation and proper integration with the specialized oncology care network.^(23,24,25)^

In clinical practice, every gynecological consultation should be viewed as an opportunity to assess the patient's eligibility for breast cancer screening and reinforce current recommendations. This approach should incorporate individualized risk stratification to determine the most appropriate screening strategy based on age, family history, genetic predisposition, breast density, and personal medical history. Shared decision-making is recommended, grounded in clear, evidence-based information regarding the benefits and potential risks of screening, including false-positive results and overdiagnosis. Communicating this information in understandable language is essential to fostering an informed decision that aligns with the patient's values and preferences.^(17,24,25)^

In addition to ordering the mammogram, the gynecologist must ensure organized clinical follow-up by documenting management decisions, providing guidance on the examination process, and monitoring results. Suspicious findings should prompt a timely referral for further diagnostic investigation to avoid delays in confirming the diagnosis and initiating treatment (Table 2). Adopting a structured care pathway that includes longitudinal patient monitoring reduces loss to follow-up and contributes to the diagnosis of breast cancer at early stages, positively impacting clinical outcomes.^(17,24,25)^

**Table 2:** BI-RADS classification: diagnostic impression, recommended management, and risk of malignancy

Category	Diagnostic impression	Recommendation	Cancer risk (%)
0	Incomplete	Additional imaging required	Incomplete evaluation
1	Normal	Annual routine exam	0
2	Benign finding	Annual routine exam	0
3	Probably benign finding	Perform early monitoring (at 6, 12, 24, and 36 months)	≤ 2
4	Suspicious finding	Proceed with investigation: perform biopsy	3%- 94%
5	Highly suspicious finding	Proceed with investigation: perform biopsy	≥ 95
6	Known biopsy-proven malignancy	Appropriate treatment	100

Source: Adapted from the Instituto Nacional de Câncer (INCA), 2024.^(26)^

## Should mammography screening begin at age 40 or 50?

Determining the age at which screening should begin remains a subject of controversy among national and international guidelines. Evidence indicates that starting at age 40 may be associated with earlier detection and a potential reduction in mortality, particularly in populations with significant incidence rates in younger age groups, as observed in Brazil.^(13)^

In this context, the historical divergence between the recommendations of medical societies and the Ministry of Health regarding the starting age for mammography screening has recently narrowed following the position taken by the National Regulatory Agency for Private Health Insurance and Plans (ANS), which now recommends screening starting at age 40. Thus, the decision to screen should continue to be based on individualized risk assessment and shared decision-making.

## Final considerations

Mammography screening is an established secondary prevention strategy with a proven impact on reducing breast cancer-specific mortality. However, its effectiveness at the population level in Brazil remains contingent upon overcoming structural challenges, including regional inequalities in access, insufficient coverage, and the absence of widely implemented organized programs. Harmonizing guidelines, based on the best available evidence and adapted to the national epidemiological profile, is essential to ensure consistency in clinical practice and the sustainability of public policies. Risk stratification and individualized management should guide decision-making, aiming to maximize benefits while minimizing potential harms associated with screening. The gynecologist plays a central role in the high-quality implementation of this strategy, conducting individual risk assessments, promoting adherence to screening, and coordinating care within the healthcare network. Strengthening this role, combined with the structured organization of screening programs, is crucial to maximizing the impact of secondary prevention on reducing breast cancer mortality in Brazil.

## Data Availability

research data are available in the article

## National Commission Specialized in Mastology of the Brazilian Federation of Gynecology and Obstetrics Associations (Febrasgo)

President:

Rosemar Macedo Sousa Rahal

Vice-president:

Felipe Pereira Zerwes

Secretary:

Clecio Enio Murta de Lucena

Members:

Iris Rabinovich
Cynthia Mara Brito Lins Pereira
Eduardo Camargo Millen
Adriana Magalhaes de Oliveira Freitas
Andrea Pires Souto Damin
Fabricio Palermo Brenelli
Francisco Pimentel Cavalcante
Guilherme Novita Garcia
Ivo Carelli Filho
Felipe Eduardo Martins de Andrade
Vilmar Marques de Oliveira
Vinicius Milani Budel
Antonio Luiz Frasson
